# Ethno-diversity within current ethno-pharmacology as part of Israeli traditional medicine – A review

**DOI:** 10.1186/1746-4269-2-4

**Published:** 2006-01-09

**Authors:** Efraim Lev

**Affiliations:** 1 Dep. of Eretz Israel Studies and School of Public Health, University of Haifa, Har Carmel, Haifa, 31905, Israel

## Abstract

The Holy Land has absorbed millions of immigrants in recent centuries: Jews from East and West, Druze, Circassians, Muslim and Christian Arabs. The land is unique and diverse in geographical location and ethnic groups, and also in its cultural characteristics, including traditional medicine and use of *materia medica*. However, these traditions have waned over the years. The young state of Israel adopted a "melting pot" approach to fashion Jews from all over the world into Israelis.

The traditional medicine and *materia medica *of different ethnic groups (Yemenite, Iranian, and Iraqi Jews) are reviewed in this paper, as well as the ethno-botanical survey (first conducted in the 1980s, covering Bedouins, Druze, Circassians, and Muslim and Christian Arabs), and the matching ethno-pharmacological survey (conducted in the late 1990s) covering the medicines sold in stores.

Present-day healers are usually not young and are believed to be the end of the chain of traditional medical knowledge. The ethno-diversity of Israel is becoming blurred; modernity prevails, and ethnic characteristics are fading. The characteristic lines of traditional medicine and *materia medica *have hardly lasted three generations.

A salient former dividing line between ethnic groups, namely their use of different medicinal substances, paradoxically becomes a bridge for conservative users of all groups and religions. Shops selling these substances have become centers for "nostalgia" and preserving the oriental heritage, traditional medicine, and medicinal substances!

## Introduction

The Land of Israel has absorbed millions of immigrants at the last several centuries. Bedouins and the Druze arrived on account of internal Middle Eastern politics in the early Ottoman period (16^th^–17^th ^centuries). Other different ethnic groups such as Bosnians and Circassians were deported from their homelands to the Holy Land by the Ottomans in the 18^th ^and 19^th ^centuries, the most important ethnic group that stayed in Israel being the Circassians. Later, in the 19^th ^and 20^th ^centuries, at the end of Ottoman rule and under the British mandate, Muslim Arabs and fewer Christian Arabs immigrated from neighboring Arab countries in parallel with Jewish immigration, because of the improvement of the economic situation and opportunities for work.

Jewish immigration to the Holy Land from Eastern Europe started in 1882, and was followed by several subsequent waves. Yemenite Jews came later, at the beginning of the 20^th ^century. Most of the next waves of immigration were the result of international political events: Jews who fled Nazi Europe, Holocaust survivors after World War II, and Jewish refugees from Arab countries, who came after the establishment of the state of Israel. These newcomers replaced Arab refugees, who left for neighboring Arab countries. Since then immigration has continued on a smaller scale and the most abundant waves have been of Jews from USSR (1970s to 1990s) and most recently Ethiopian Jews (1990s) and Jews from Argentina (2002–2004).

These events, which are not the main subject of the article, along with the religious-political history of the Holy Land, have created a unique mosaic of a wide range of ethnic and religious groups. Their existence in such a small land caused unfortunate and familiar disputes, though from a purely academic point of view this circumstance creates an opportunity for research in many fields, among which we can cite anthropology and ethno-pharmacology.

The land itself is unique, in its natural diversity due to its geographical location at the meeting point of three continents (Asia, Africa, and Europe), the desert and the Mediterranean Sea, and the Rift Valley. Different climatic, phyto-geographic, and zoo-geographic zones converge here, creating great biological multi-diversity [1]. The region served as an important crossroads for international trade from early antiquity, between Mesopotamia and Egypt, and the East (Asia) and West (Europe); this added to the diversity of the materials used as medicines [2].

The ethnic groups which compose this impressive mosaic in the Holy Land retained to varying degrees their languages, religions, traditional food, clothing, ceremonial customs, and traditional medicine, including the use of special medicinal materials. However, the diversity of medicinal substances in this land is not a new phenomenon; evidence of the existence of an remarkable inventory and international trade, caused by factors similar to those outlined, can be found in the Bible, in classical literature, in Arabic sources, and in other contemporary historical sources. The diversity of the medieval inventory of *materia medica *has also been researched, and it displays notable variety [3].

However, cultural traditions have dwindled over the years, especially in the State of Israel. The young state contributed heavily to cultural homogenization, by imposing an unwritten rule of the "melting pot" in order to transform Jews from all over the world into new, modern Jews – Israelis [4]. This approach has accelerated the natural process of cultural assimilation during the last 55 years.

The unique, distinctive ethnic mosaic of Israel is blurring rapidly, succumbing to processes of modernity and globalization. This article sets out to study, understand, record, hence preserve, ethno-diversity from an unusual angle, namely traditional medicine in general and the medical uses of natural materials in particular.

## Mapping the mosaic: a cultural, numerical, and geographical description of the ethnic groups

The population of the state of Israel in 2004 was approximately 6,750,000 people, most of whom (91%) live in towns and pursue a highly Westernized and modern life style. The majority consists of 5,450,000 (81%) Jews; there are several minority groups, mainly Muslims (1,000,000) and Christians (142,000) [5].

### Jewish sector

#### Eastern Europe, America, and Western Europe

Hundreds of thousands of Israeli Jews, most of whom arrived from eastern European countries, have immigrated to Israel over the last 70 years; their traditional culture and habits mostly do not include special or unique traditional medicine [6].

#### India

More than 23,000 Jews of Indian origin had immigrated to Israel by 1970. They are a small, united community, separated into two main subgroups according to their place of origin in India [6]. They preserve their culture, but no data regarding special traditional medicine in Israel have been published.

#### Yemen

Yemeni Jews arrived in Israel in two waves, one at the beginning of the 20^th ^century and the other in the 1950s. It is estimated that altogether about 45,000 Jews from Yemen immigrated to Israel [6]. A few more families arrived in the late 1990s; anecdotally the interesting and vanishing tradition of eating locusts was recorded from them. Since they were a small community (and for other anthropological reasons) they have maintained their culture and tradition, including traditional medicine, better than other ethnic groups. Yemeni Jews are known for their good health, longevity, and, by contrast, specific diseases.

#### Iraq

Most of Iraqi Jewry immigrated to Israel between 1948 and 1951; they numbered about 123,500 people. They came as an organized community and were mainly of urban origin, and merged well into Israeli society [6]. Yet they retained their language and some cultural characteristics and traditions, including traditional medicine.

#### Iran

More than 55,000 Jews have immigrated to Israel over the years, mainly between 1948 and 1968. Though many others emigrated to the USA, this community has kept its tradition better than others [6]. Specialized shops dealing in spices and medicinal substances (with original names different from those of other ethnic groups) still do business in Tel Aviv and Jerusalem [7].

#### Kurds

It is estimated that more than 32,000 Jews of Kurdish origin have immigrated to Israel (from both Iraq and Iran). Most of them came from agricultural areas and small towns [6]. This ethnic group was scattered over a wide territory in their land of origin and its members did not engage in well organized communal activity after their arrival in Israel.

#### Morocco

More than 200,000 Moroccan Jews immigrated to Israel during the 1950s and 1960s. This was the biggest North African ethnic group ever to arrive, although tens of thousands of other Moroccan Jews, including most of their religious and communal leaders, went to France and Canada [6]. Several traditional characteristics were maintained for several decades, but no proper study of their traditional medicine has ever been conducted. The data are scattered in general literature and books on daily life [8].

#### Other countries of North Africa

About 100,000 Jews from elsewhere in North African Jews entered Israel between 1948 and 1970, mainly from Libya (30,000), Tunisia (40,000), and Algeria (30,000). A large part of each community, including leaders and wealthy families, emigrated to France and other locations [6].

#### Middle Eastern Arab countries

More than 45,000 Jews arrived in Israel (1948–1968) from Syria (5,000), Lebanon (5,000), and Egypt (35,000) [6].

#### Ethiopia

Tens of thousands of Jews have entered Israel from Ethiopia since the early 1980s, in a few large waves and secretly as a steady flow. This unique ethnic group represents the most recent influx of Jews to Israel. Their remote origin in Central East Africa forced this group to keep and preserve their tradition for centuries due to their isolation, their strong communal sense, and their powerful awareness of their Jewish religion. Their circumstances have been studied from diverse angles. Besides interesting social and religious traditions, they brought to Israel an entire culture of unique foods, spices, traditional medicine, and medicinal substances [9,10].

Oriental Jews of Moroccan, Middle Eastern, and North African origin are the best customers at shops selling spices and medicinal substances in cities such as Jerusalem, Tel Aviv, Haifa, Acre, Ramlah, and Lod (personal observations).

### Minority groups

Minority groups tend to maintain traditions and culture better than majority groups. Such seems to be the case in the State of Israel, although modernization and globalization are not passing the younger generation of these groups by. The "global melting pot" seems to include minority groups whose youth tend to prefer general knowledge more than the traditional. Practical and theoretical knowledge of plants and animals and their uses tends to be poor among Jewish students as well as among Muslims, Christians, Druze, and Circassians; very few of any group show any interest in studying it at all! [Personal observations and oral communication with other scholars.]

#### Arab Muslims

These form the biggest minority group in Israel, numbering about one million (16% of the total population). This ethnic group is geographically concentrated in two major centers, Galilee and north central Israel [5]. The Muslims live in towns, cities, and villages.

#### Bedouins

Muslim by religion, the Bedouin ethnic group comprises nomadic tribes from neighboring Arab countries, mainly of Arabian origin. Most of the Bedouin tribes' ancestors reached Israel around the 7^th ^century CE. Today they live mainly in two centers: the Negev desert (85,000) and a much smaller center in the Galilee [11].

#### Arab Christians

The members of this ethnic group (115,000) constitute different communities according to their religious affiliation and membership of congregations [5]. Most of the Arab Christians in Israel form urban communities in Haifa and Nazareth, and the rest reside in adjacent small towns and villages.

#### Druze

This minority community in Israel (100,000) is a close-knit monotheistic religious sect that broke away from Islam in Egypt in the 11^th ^century. In Israel the Druze live in 20 villages in the Golan, Galilee, and Mount Carmel. As a closed sect, they tend to keep and preserve their culture and traditional habits [5].

#### Circassians

These are a small minority community (3500), Muslims of Asian origin, who were transported to the Holy Land by the Ottomans at the 18^th ^century. Today they live in two villages in Galilee [5]. They use local non-cultivated medicinal plants collected in the wild, as well as cultivated plants, in the manner of their Arab neighbors. No use of special plant species had been recorded.

## Current research overview

During the last 40 years several ethno-botanical and ethno-pharmacological studies have been conducted with the aim of learning about and conserving traditional medicine and the medicinal uses of plants, animals, and minerals by different ethnic groups. The studies were done by different scholars from a wide range of academic disciplines such as medicine, anthropology, pharmacology, botany, and folklore. Because the data extend over four decades, and were gathered by different methods and skills, their analysis and synchronization remain a difficult and even hazardous task, although it is possible. Brief descriptions and evaluations of the main research studies are listed below (available quantitative data are shown in table 1).

**Table 1:** Lists of *materia medica *compiled among Israeli ethnic groups and minorities

Ethic Group	Plants	Animals	Inorganic	Total	Reference
**Yemenite Jews**	151	21	10	182	[18]
**Iranian Jews**	65	3	2	70	[21]
**Iraqi Jews**	150	16	15	181	[20]
**Bedouins**	193	42	15	250	[17]

• The data were gathered from diverse sources on different academic levels of research.

• The only group whose members still live an active life in the wild are the Bedouins, so the lists of plants and animals recorded as used in their medicine are larger than in other groups.

### An ethno-botanical survey

was conducted in Israel in the 1980s and covered about 100 informants belonging to most minority ethnic groups (Arab Muslims and Christians, Druze, Bedouins, and Circassians). This survey yielded information on the medicinal uses of some 447 plants [12,13]. Prior to this field survey a few smaller-scale studies had been conducted over the years [14]. Several regional surveys have been published as well [11,15,16].

### Bedouins

The traditional medicine of the Bedouins in southern Israel was investigated by A. Abu-Rabi'a. Himself a Bedouin born in the Negev, he interviewed dozens of informants, recording the use of 193 medicinal plants, wild as well as cultivated [17].

### Yemeni Jews

A member of this ethnic group, Y. Raiany conducted research on their traditional medicine and *materia medica *for an MSc thesis in pharmacology, interviewing 57 informants. This unpublished work, recording 151 medicinal plants, which was written in the early 1960s [18], has become an important source, together with newly published book on the special diet and traditional medicine of Yemeni Jews [19].

### Iraqi Jews

A. Ben-Ya'akov researched Iraqi Jews' traditional medicine and presented it in a two-volume book. It contains a vast amount of information on the traditional medicine of this ethnic group in Iraq, the names of healers, and the techniques, methods, and medicinal materials used by Iraqi Jews in Iraq and in Israel. He interviewed scores of healers, recorded booklets of some of the most important of them, and reported on the use of 150 medicinal plants [20].

### Persian Jews

The Persian (Iranian) Jews have kept the habit of using their special traditional *materia medica *better than other ethnic groups in Israel. The Persian materials, some of which are unique and used only by them, have Persian names and are sold in several instances in specialist shops in Tel Aviv and Jerusalem [7]. A. Pikel conducted and published research on the traditional medicine of the Iranian Jews in Israel, based on interviews with a few remaining healers, describing the substances (65 medicinal plants) and their uses [21].

### Ethiopia

A study on their *materia medica *is being conducted in Israel. We found it extremely hard to obtain information from the healers for fear of competition and other reasons. Israeli-born students were not acceptable as interviewers; students of Ethiopian origin did better, but still not well enough – they recorded only the basic materials (personal observation). Research of the medicinal substances of the Ethiopian Jewish community in Israel will apply qualitative methods based on accepted ethno-botanical and ethno-pharmacological principles [22,23]. We intend to interview twenty prominent elderly sages of this ethnic group who are known as healers, or are sons of well known healers. Ethiopia has a unique bio-diversity, and a great many medicinal plants used and traded extensively in the past have been recorded among its wild flora (e.g., kinds of myrrh and frankincense).

### The ethno-pharmacological survey

The survey of the late 1990s complemented the ethno-botanical survey of the 1980s. It covered medicinal materials purchased at stores and not available in the wild. The survey made it possible to prepare a summary list of all the materials in use today in Israeli traditional medicine. This list opens a window to a whole and usually hidden world of medicine, traditions, and customs that still persist in Israel and neighboring countries [7,24]. It includes information regarding medicinal substances of all ethnic and religious groups in Israel. The numerical data are summarized in Table 2.

**Table 2:** Quantitative data on *materia medica *of the Land of Israel (ethno-pharmacological market survey)

Origin	No. of materials	Percentage
**Animals**	20	6.5
**Inorganic**	19	6.1
**Others**	7	2.3
**Plants**	264	85.1
**Total**	310	100

Missing from the list may be wild plants known to be used in folk medicine today, as they were used in the past. These plants are still gathered by the traditional healers or by the patients themselves in open spaces, and are not regularly sold on the markets or at spice and drug vendors' stores. Other ethno-botanical studies examined some of these plants and their uses among the diverse communities and minorities in Israel [12]. The combined results of our survey and those of the ethno-botanical surveys provide a reliable picture of the totality of folk medicinal materials in use among the many and varied religious and ethnic populations in Israel. The researchers who conducted the ethno-botanical survey published a comprehensive list of the medicinal plants of Israel, including wild flora; that list contains 447 species of plants [13]. Together, the data yield a theoretical (potential) inventory of 629 materials (table 3).

**Table 3:** Quantitative data on *Materia medica *of the Land of Israel (including the ethno-botanical survey)

Origin	Wild/Local	%	Domesticated local	%	Imported	%	Total	%
**Animals**	8	40	3	15	9	45	20	3.5
**Inorganic**	2	10.5	-	-	17	89.5	19	3.0
**Others**	2	28.6	-	-	5	71.4	7	1.1
**Plants**	447	76.9	28	4.8	106	18.3	581	92.4
**Total**	459	73.2	31	4.9	137	21.9	627	100

## Discussion

### The *materia medica*

#### Plants

Plants yield most of the medicinal materials, a persistent feature evident in medical literature throughout history [3,24]. The vast majority of plants sold in the stores as popular remedies are cultivated, and only a few are wild. This is understandable, particularly considering the argument that wild plants are collected privately and used widely on a non-commercial basis by the patients or folk healers; this is why they were not found in the shops or included in our lists. However, the greater part of the local medicinal plants (447) are wild, and far fewer (28) are cultivated: almonds, apple. beet, carrot, eggplant, garlic, lettuce, olive, onion, pomegranate, quince, radish, and spinach.

Geographical location is crucial issue in the case of the wild plants, mainly due to the unique differences in, hence bio-diversity of, climate (temperature, humidity, wind, rainfall), elevation, soil, and other abiotic characteristics. The wild flora of the Land of Israel's southern (Negev) and eastern parts (Rift Valley and Judean desert) consists of desert and tropical plants, while the northern part (Carmel, Galilee, and Golan Heights) is more replete with Mediterranean species.

The Druze of the Golan Heights, Mount Carmel, and Galilee, as well as the Bedouins and the Arab Muslims and Christians of the Galilee, mainly use common wild plants such as knee-holly (*Ruscus aculeatus *L.), laurel (*Laurus nobilis *L.), lemon balm (*Melissa officinalis *L.), rue (*Ruta chalepensis *L.), sage (*Salvia fruticosa *L.), and wild majoram (*Origanum syriacum* L.) [11,12,15]. These are wholly different from the desert plants that are used regularly by the Bedouins of the Negev and the Judean Desert, such as ben tree (*Moringa peregrina *L.), Syrian rue (*Peganum harmala *L.), toothbrush tree (*Salvadora persica *L.), Judean wormwood (*Artemisia judaica *L.), and white wormwood (*Artemisia herba-alba *Asso.) [11,13,17]. A few plants that grow all over the country are used by all religious and ethnic groups in northern as well as southern Israel and the Middle East; two examples are the castor oil plant (*Ricinus communis *L.) and Christ thorn (*Ziziphus spina-christi *Willd.) [25]. There are very few inter-ethnic differences in the use of wild medicinal plants in Israel apart from those due to climate and geography. An example of such a difference in use of cultivated plants is Jews mallow (*Corchorus olitorius *L.): it is eaten and used for medicinal purposes by Muslims, Christians, and Jews of diverse origin, mainly Yemen, Egypt and Iraq. But due to religious laws it is not consumed or used medicinally by the Druze [3,24,26].

In this research, spices and condiments are included as medicinal substances, in addition to their important role as foodstuffs. Almost all of them are known as medicinal plants in different cultures. The different Israeli purchasers and consumers of medicinal plants buy spices and condiments for their medicinal properties, their nutrition, and in some cases even as incense (e.g., cinnamon).

#### Animals

The use of animal organs and products did not flourish towards the end of the second millennium; this is understandable in light of changes of patterns of medical practice among consumers of traditional medicine, and perhaps also in connection with changes in moral standards and attitudes to animals and their use in medicine [24,27]. Nine substances are imported into Israel; however, the data clearly show that most (8) of the local medicinal materials obtained from animals originate from wild species, and only a minority (3) from domesticated species. A possible explanation is the massive stock of animal products in the general stores; also, patients and folk healers may prefer to buy products such as cheese, milk, meat, eggs, chicken, and animal parts, which are used in folk medicine, in non-specialist shops, where good quality and freshness is promised. Therefore, the folk remedy vendors in the shops and markets sell mainly traditional products derived from wild species and special animal products unavailable in regular shops, such as wax and hard dry cheese. Because most of the population in Israel is urban and far from wild animals, and because the nature preservation laws are strictly enforced, products from wild animals are found in only few shops [28].

Geographical location is crucial in this case too; for example, the Sinai desert Bedouins make medicinal use of several Red Sea animals [29].

#### Inorganic

Inorganic material likewise is used much less than plants. The explanation for this discrepancy seems to lie in the users' perception that some inorganic materials used in the past are poisonous. We may also mention that in the past too, in all cultures, inorganic substances formed only a small part of the inventory of medicinal materials [24,30-32].

#### Origin

The great majority of the materials used in folk medicine in Israel are of local origin, very few being brought in from other countries. A comparison makes clear that wild and cultivated plants, for eating and for use in folk medicine, are collected in open areas, fields, and plantations by patients, healers, and folk medicine practitioners. These plants did not appear in the survey of shops and markets, but we made a point of not overlooking their use. Recall also that a large proportion of the imported plants that appear in the list are spices originating in Asia, as well as local and imported condiments. These are used by regular customers for seasoning and cooking, but as noted in many cases are also used as medicinal plants in local ethnic folk medicine.

#### Past versus present

From analysis of the list of *materia medica *we can learn sources of the various materials and past and the present trade routes. We can also test claims arising from historical research that a local medical and pharmacological tradition existed in Israel and its surroundings, and the area served as a junction for the transit of medicinal materials and as a base for exporting many other materials. Information on the inventory of the *materia medica *used in the Holy Land in the Middle Ages, prior to the waves of immigration, is available [3,26]. So is a rare record of a visit to the markets by the Swiss physician Dr. Tobler at the mid-19^th ^century [33,34].

#### Israel compared with neighboring countries

Supporting information regarding the traditional uses of natural medicinal substances, may be derived from ethno-botanical and ethno-pharmacological surveys conducted in neighboring countries such as Egypt [35-37], Jordan [38], Syria [39,40], Lebanon [41], and other Middle Eastern countries [42] (see Table 4).

**Table 4:** Lists of *materia medica *compiled in some Middle Eastern countries

Country	Plant	Animal	Inorganic	Other	Total	Source
**Syria**	189	11	31	13	244	[39, 40]
**Jordan**	236	30	29	9	304	[38]
**Israel**	264	20	18	7	309	[24, 26]

Other surveys have been conducted in the countries of origin of some Jewish ethnic groups mentioned in this study such as Iraq [43,44], Iran [44], Libya [45], Egypt and Morocco [42], and Yemen [39]. These helped us to identify the special materials and better understand their inventory and regional uses.

#### Core inventory

Horizontal study and analysis of the *materia medica *of all the different ethnic and religious groups in Israel [7,17-21,24] revealed a core inventory (the most important and used substances of all Israeli ethnic and religious groups, according to market and literature surveys), which we believe satisfies the basic needs in traditional medicine of any Middle Eastern ethnic group [38-40,42-44]. It consists of about 40 plant materials, five animal, and five inorganic. Each ethnic, religious, or geographic group has a further 100–150 general materials and a dozen special materials, mainly plants, usually of local origin, which are uniquely its own. In most cases these materials bear special local names! The core inventory is alike for most Middle Eastern ethnic groups, so its items will be available in most shops. It is presented in Table 5.

**Table 5:** Reconstructed core inventory of Middle Eastern *materia medica*Inorganic

Name	Main Uses		
Alum	General tonic, reduces liver size, disinfection		
Citric acid	Digestive aid		
Galena	Cosmetic, eye diseases		
Lead	Skin diseases, evil eye		
Sulfur	Fertility, skin diseases		

**Animals**			

**Scientific Name**	**English Name**	**Part or Product**	**Main Uses**

*Apis mellifera *L.	Honey bee	Wax & Honey	Purgative, reduces eye inflammations, sore throat, burns, coughs
*Physeter catodon *L.	Ambergris	Secretion	Reinforces potency, kidneys, and General
*Scincus scincus *L.	Medical skink	Dry body, Skin	Fertility of men and women
*Sepia officinalis *L.	Cuttle fish	Skeleton	Skin diseases, mania

**Plants**			

**Scientific Name**	**English Name**	**Part or Product**	**Main Uses**

*Aloe vera *L.	Aloe	Juice	Skin diseases, heals wounds, purgative
*Alpinia galanga *willd.	Galingale	Root	Cleans digestive system, reproductive system problems
*Artemisia herba-alba *Asso.	Wormwood	Foliage	Asthma, sore throat
*Boswellia carteri *Birdw.	Frankincense	Resin	Flatulence, heart diseases, general tonic
*Cassia acutifolia *Delile.	Senna	Leaf	Purgative
*Cinnamomum zeylanicum *Nees.	Cinnamon	Bark	Cleans the female genital system after childbirth
*Coriandrum sativum *L.	Coriander	Seed	Reduces sugar, stomachache
*Crocus sativus *L.	Saffron	Stigma	Sore throat, blood pressure
*Cuminum cyminum *L.	Cumin	Seed	Flatulence
*Curcuma longa *L.	Turmeric	Root	Disinfects sores
*Elettaria cardamomum *White & Maton	Cardamom	Fruit	Blood sugar, stomach acidity
*Eugenia caryophyllata *Thumb.	Clove	Flower	Local anesthetic, general remedy
*Foeniculum vulgare *Mill.	Fennel	Seed	Stomachache, intestinal diseases
*Glycyrrhiza glabra *L.	Liquorice	Root	Heartburn, coughs, blood cleansing
*Laurus nobilis *L.	Laurel	Leaf	Arthritis, sugar in blood
*Lawsonia inermis *L.	Henna	Leaf	Wound healing, hemorrhages, fungi
*Lepidium sativum *L.	Garden-cress	Seed	Stomachache, hair strengthening, aphrodisiac
*Linum usitatissimum *L.	Flax	Seed	Hormonal regulation, calcium supplementation
*Matricaria aurea *Boiss.	Chamomile	Flower	Respiratory canals, stomachache
*Mentha spicata *L.	Mint	Branch	Flatulence, acidity neutralization
*Myristica fragrans *Houtt.	Nutmeg, mace	Seeds and fruit peels	Intoxicant, aphrodisiac, painkiller
*Nigella sativa *L.	Black cumin	Seed	Dizziness, sugar in blood
*Olea europaea *L.	Olive tree	Oil	Skin diseases, many medications
*Peganum harmala *L.	Syrian rue	Seed	Tonic, heart diseases, sugar in blood
*Petroselinum sativum *Hoffm.	Parsley	Seed	Kidney stones, impotence
*Pimenta officinalis *Lindl.	Pimento	Fruit	Stomachache, flatulence
*Pimpinella anisum *L.	Anise	Seed	Stomachache, aphrodisiac
*Pinus pinea *L.	Stone-pine	Seed	Tonic
*Piper nigrum *L.	White pepper	Fruit	Coughs, reinforces stomach
*Pistacia lentiscus *L.	Mastic tree	Resin	Heartburn, soothes stomach
*Prunus mahaleb *L.	Perfumed cherry	Seed	Digestive system, children's pains, flatulence
*Rhus coriaria *L.	Tanning sumach	Fruit	Perspiration stimulant, diarrhea, cholesterol reduction
*Rosa sp.*	Rose	Water, flowers, fruits	Perfume production, cleanses facial skin
*Rosmarinus officinalis *L.	Rosemary	Foliage	Kidney stones, sugar in blood
*Saccharum officinarum *L.	Sugar cane	Juice	Tranquilizer, tonic, respiratory canals
*Salvia fruticosa *L.	Sage	Leaf	Hemorrhages, intestinal diseases and pains
*Sinapis alba *L.	Mustard	Seed	Pains and infections
*Tamarindus indica *L.	Indian date	Fruit	Cleanses blood and stimulates circulation
*Trigonella foenum-graecum *L.	Fenugreek	Seed	Sugar reduction, diuretic
*Zingiber officinale *Rosc.	Ginger	Root	Flatulence, digestive system, impotence

### Geographical aspects

#### International geo-diversity

International geographical location is an important factor, especially considering the different ethnic Jewish groups. Each group, according to its geographical origin, brought to Israel the customs of using local medicinal plants which were common there. Thus the Jews that came from Syria and Lebanon did not bring with them ways of using different substances from those that prevailed among the local Arab population in Israel. But Jews who immigrated from more distant parts, such as Iran, Iraq, Yemen, India, Ethiopia, or North Africa, introduced their own ways of using local substances or those imported from their homeland, the latter often being exotic. In general, they bought their special medicinal substances as well as their traditional spices and condiments at the town markets of their new home, Israel. However, during the 1950s and 1960s some of the newcomers, in particular the healers, might have gathered some local wild plants (non-cultivated) near their dwelling places. These could be the borderland in the north or south of Israel, where the development towns were located to absorb the mass immigration, or farms and small villages where they were placed by the immigration authorities. Since the 1970s this phenomena has become very rare, and no research has recorded new immigrants of the last few decades such as Ethiopian or Yemenite Jews collecting wild plants for medicinal uses.

However, in the markets of big cities and towns such as Jerusalem, Tel Aviv, Ramlah, Lod, Tiberias, and Haifa, and in other small towns, where groups of new immigrants of particular origin reside, specialist shops do exist, selling ethnic foods, spices, and medicinal substances, especially plants imported from Ethiopia.

We have chosen to enumerate here, as an example, some of the special, and common medicinal plants of the Yemeni, Iranian, Iraqi, and Ethiopian Jews. The aim is to help the reader understand the characteristic features of these ethnic groups, and to highlight the differences among them, in contrast to the similarities that emerge from the core inventory presented in table 5.

#### Yemeni Jews

asafetida (*Ferula assa-foetida L.*), Christ thorn (*Ziziphus spina-christi Willd.*), common jujube (*Ziziphus jujube Lam.*), pine tar (*Pinus sp.*), katarn (*Cedrus sp.*) purging cassia (*Cassia fistula L.*), screw pine (*Pandanus odoratissimus L.*) [18,19].

#### Iranian Jews

alhagi manna (*Alahgi graecorum* Medic.), areca nut (*Areca catechu* L.), basil (*Ocimum pilsum* L.), descurainia (*Descurainia sophia* (L.) Webb.), lallemantia (*Lallemantia royleana* (Wall. ex Benth.) Benth.), matchbox bean (*Entada* scandens Benth.), white pilsum (*Ocimum canum* Sims) [7,21].

#### Iraqi Jews

bdellium tree (*Commiphora mukul *Engl.), black myrobalan (*Terminalia chebula *Retr.), sweet lemon tree (*Citrus limetta *R.), sweet violet (*Viola odorata *L.), yellow myrobalan (*Terminalia citrina *Roxb.), white willow (*Salix alba *L.) [20].

#### Ethiopian Jews

black mustard (*Brassica nigra *Koch.), henna (*Lawsonia inermis *L.), Madagascar cardamums (*Amomum angustifolium *Sonner.), Niger seed (*Guizotia abyssinica *(L.f.) Cass.), mustard collard (*Brassica carinata *L.), spinach rhubarb (*Rumex abyssinicus *Jacq.) [46,47].

#### The geography of trade in *materia medica*

The trading habits of the drug sellers reflect the actual demands of the community of clients they serve. In-depth investigation shows that the relationship and affiliation with suppliers in Arab countries is a matter of geography, whose chart is a relic of the pre-modern political map. The only traditional drug seller working in Nazareth has family ties in Jordan, and used to buy special materials, mainly of animal and mineral origin (which are rarely imported into Israel by Jewish dealers due to low demand and strict laws), in the city of Shechem (Nablus). Since the peace agreement between Israel and Jordan was signed, he has imported them directly; his preferred suppliers are in the northern cities and towns of Jordan. On the map of the state of Israel and its Arab neighbors, the family and business connections and affiliations in many fields, including imports of *materia medica*, are quite evident [Shechem & Jenin – northern Jordan and Amman; Jerusalem & Ramallah – Amman; Hebron – Egypt] [24]. This phenomenon is not unique to Israel: in an ethno-pharmacological survey conducted in Jordan we discovered a similar geo-diverse relationship with the other Arab neighboring countries. This system affects the types of substances in the shops, and reflects the impact of these countries on the local inhabitants. Drug sellers in northern Jordanian cities have ties with Syria and Lebanon and stock special Syrian and Lebanese *materia medica*, those in the central and eastern cities sell to Iraq and Iran, and those in the southern cities deal with Egypt, Yemen, and Saudi Arabia [38].

### The practice and business of traditional medicine

#### Present-day healers

Clearly, present-day healers, regardless of origin or religion, are not young, and rarely are their children willing to learn from them! In my opinion, the current generation of traditional healers in most of the aforementioned ethnic groups represents the end of a chain of medical knowledge that started in prehistory. In today's world, where modern Western medicine dominates, it is the task of the ethno-botanist and ethno-pharmacologist to collect the remnants of information and knowledge and conserve them for use in the future.

#### Healing methods

The healing methods are varied (diets, aromatic baths, medicinal potions, pills, ointments, inhaling aromatic plants, steam, etc.) and are not the topic of this article. In most cases, though, they are based on medieval Arab medicine [48-50] which in turn was based on classical Hippocratic-Galenic methods and theory. The uses of the *materia medica *and the pharmacology derive from Arabic sources. Examples are books by the medieval Jewish (Karaite) physician Ibn Abi 'l-Bayan, *al-Dustur al-Bimaristani *(The Hospital Handbook) [51] and by al-Kohen al-'Attar, *Minhaj al-Dukkan *(The store guide) [52], and classical sources such as Dioscorides [53]. One historical case-study of medieval medical theories practiced in the Levant revealed traces of the use of the doctrine of signature [54]. Interestingly, evidence of similar usage has been detected in present-day traditional applications of medicinal plants in Israel [55]. A survey made on the use of the Solanaceae species in traditional medicine in Israel clearly indicated that healers deliberately avoided the use of narcotic agents found in this family [56]; another survey examined the role of Labiatae among medicinal plants in Israel [57].

#### Present-day traditional drug vendors

Another phenomenon is that the drug shops are usually operated by young people (30–50), sometimes the third generation of drug sellers; yet their education is not of a high level and they lack crucial knowledge. When they are asked a specific question, they look at one of the above-mentioned books or call their elderly parents who consult the book at home! I have seen several cases of a seller who learned about the medical use of one of the plants in his shop from a client, and conveyed it to the next customer when asked about its uses.

In the special inter-religious/ethnic relationship in the *materia medica *trade it is common, for example, to see Arab Muslim or Christian dealers from Nazareth, Acre, or Jerusalem buying imported Indian products from a shop in Tel Aviv owned by a Jewish Yemeni. Sometimes they will sell the same products to Ethiopian or Moroccan Jews, as well as Arab Muslim and Christian women, in their shops the next day.

#### Shops selling traditional *materia medica*

The number of shops in the Arab sector and the Jewish sector has been falling steeply, although the number of modern shops selling spices and "modern" alternative medicinal materials is increasing (in most cases they sell identical substances, but in fancier packaging). The large and well established shops have survived this process by selling other goods besides spices, grains, and medicinal substances. Lately, as mentioned above, a few new specialist shops have opened near neighborhoods with a high percentage of new immigrant Ethiopian Jews. These shops supply food, spices and *materia medica*, mostly imported from Ethiopia by specialized traders [46,47].

### The "customers"

#### The customers

Customers are from all religious and ethnic groups, though traditionally they would look for a shop holding stock comprising the special material of their own ethnic group.

#### Minorities sector

No major differences were found in the traditional medicine habits of the different minority religions (Muslim, Christian, and Druze). However, diversity enhanced by geographical location (geo-diversity) is a common feature, mainly expressed in gathering plants in the wild.

#### Jewish sector

The inventories of the Jewish ethnic groups when compared with surveys made in their countries of origin showed, not surprisingly, that the Jewish communities practice similar traditional medicine, both in healing methods and medicinal substances. The explanation lies in the history of the region: in the Middle Ages the Islamic world, where for centuries Syria, Iraq, and Egypt consecutively constituted the center, Jews and Muslims maintained very close relations, including business partnerships. Many Jews were physicians, pharmacists, drug sellers, and potion makers, and they ran international businesses, trading mainly in *materia medica *[58]. Jews treated the rulers, sometimes the highest religious authorities of the country; they were admired by the Muslim physicians and wrote some of the most important and widely read medical books. These were of immense importance, and as mentioned, are used by Muslim drug vendors in Israel and the Arab countries to the present day [37]. Accordingly, the hundreds of thousands of Jewish refugees and new immigrants to Israel from the Arab world satisfied their basic demand for their familiar *materia medica *at Arab shops. At first this occurred mainly in the big cities, where Arabs lived alongside Jews, for example, Acre, Beer Sheba, Jerusalem, Nazareth, Lod, Ramlah, and Tel Aviv-Jaffa. Later, new shops were opened by members of the ethnic group, some of whom traded with their home countries, (in many cases hostile to the state of Israel) in order to supply the demand for special ethnic materials.

### General aspects

#### "Melting pot" results

Over the years modernization, globalization, and inter-communal marriage have blurred the main ethnic characteristics and differences such as appearance (hair, skin, and eye color), language (accent, vocalization), and customs (food, religious ceremonies). The typical features of traditional medicine and the inventory of *materia medica *have hardly lasted three generations in modern Israel. Here is one example from my experience: young Ethiopian students who were born in Israel have no knowledge of, nor any desire to learn, the traditional medicine of their ethnic group. Moreover, some of them can hardly communicate with their grandparents due to lack of adequate language abilities (personal observations).

#### Recording traditional medicine and *materia medica*

Several ethnic groups have not been studied, and their unique knowledge of traditional medicine and medicinal substances has not been recorded. These include mainly Jews from Morocco, Libya, Algeria, Syria, Lebanon, Egypt, India, Ethiopia, and other eastern parts of the world. With the help of academics these communities should make a last-ditch effort to preserve their knowledge for future generations.

#### Demands and availability

Inter-ethnic and religious borders are fading rapidly with time; many medicinal materials described in the books no longer exist on the market. The cause is economic: without real demand, the drug vendors will not make any effort to buy these substances in Israel or abroad. The same picture is elicited from the Cairo Genizah in letters of Jewish traders in *materia medica*. They would exchange information, mainly those at the great seaports around the Mediterranean, about demand and prices, and act accordingly [58].

The borders of Israel, Jordan, Egypt, and Morocco are open today, so theoretically it is possible buy, indirectly, any desired medicinal substances from any Arab country. However, buying exotic and expensive substances bears unnecessary financial risks – especially in the present-day Middle East, which is unlike modern Europe where the border-crossing is unhindered. Anyone entering any Middle Eastern country is subjected to stringent customs and security checks. Therefore, the market in special products from Morocco, Iran, and lately Ethiopia, which flourishes today, will dwindle markedly in the next few years as the rising generation loses interest in such products.

## Conclusion

The study of folk medicinal materials available in shops uncovered the remains of ancient medical practices that still exist in traditional societies and ethnic groups in Israel and in various other countries and cultures around the world. Due to its geo-diversity, bio-diversity, and ethno-diversity, the Land of Israel is an in-situ laboratory for a variety of processes that are studied by scholars of different fields, and should continue to be studied by more. The ethno-pharmacological surveys conducted in the Middle Eastern countries in the last 15 years are important. They recorded the *materia medica*, medicinal knowledge, and actual demand at the time they were made. Information on wild medicinal plants was gathered in a few ethno-botanical surveys.

Differences between the traditional medicine of various ethnic groups, slight as they are, still exist, and were presented above in the sense of medicinal plants used uniquely by them according to their origin (in the case of the different Jewish ethnic groups) or subsequently, in the case of local Arab inhabitants, according to their geographic (latitude) locations. Similarity among these ethnic groups lies first of all in the use of the same basic group of medicinal substances, presented above as the "core inventory" of *materia medica*. It also features in the sense of using similar healing techniques (Hippocratic-Galenic, improved by the Arabs), the importance of beliefs in saints, magic, admiration of holy graves or trees, use of amulets, etc.

However, the main traits of traditional medicine and *materia medica *appear to have hardly survived three generations. At the very start of the third millennium only a small minority of the population of each ethnic group, in the Jewish sector as well as in the Muslim and Christian ones, practice traditional medicine at all. We can assess that the present users as well as the healers are mostly old people (70–80 years old) and their second generation (50–70 years old). Member of the youngest generation, in most cases, ignore or are even ashamed of these channels of healing and prefer to use modern medical services.

An interesting issue, however, is that one of the most distinct former dividing lines between ethnic groups, namely the use of different medicinal substances, has paradoxically become a bridge linking conservative users of all groups and religions. Shops selling these substances have nowadays become centers for "nostalgia" and the preservation of the eastern heritage, folklore, food, spices, traditional medicine, and medicinal substances!
